# Supplementary material for: The Combined Effect of Acute Interval and Cognitive Training on Visual-Spatial Abilities in Women: Preliminary Insights for Health Promotion
Source: Int J Environ Res Public Health. 2025 Oct 5;22(10):1524. doi: 10.3390/ijerph22101524 (PMC12563624; doi:10.3390/ijerph22101524)
Supplement: Supplementary file 1 [file ijerph-22-01524-s001.zip › S1_Cognitive training.pdf]

CODICE SOGGETTO: ________________________________ DATA: ___________________

Le figure sottostanti le consegne sono degli esempi. Gli esercizi che dovrà svolgere sono numerati (Esercizio 1., Esercizio 2, etc.)

RIBALTAMENTO

Consegna

Le verranno presentate delle figure geometriche con delle caselle al proprio interno, alcune bianche e altre blu (fig. A)
Immagini che questa forma sia fatta di plastica trasparente, in modo che le caselle siano visibili anche sul retro.
Immagini ora di capovolgere la forma nelle direzioni indicate dalle frecce (fig. B).
Disegni nelle griglie finali (fig. C) la posizione in cui verranno a trovarsi le caselle blu dopo i ribaltamenti sopradescritti.
Inizi a disegnare la risposta soltanto dopo che si è formato nella mente l'immagine della posizione finale della forma e delle sue caselle.

fig. A

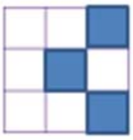

fig. B

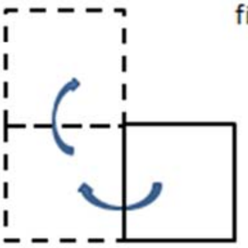

fig. C

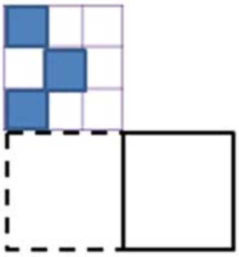

Esercizio 1.

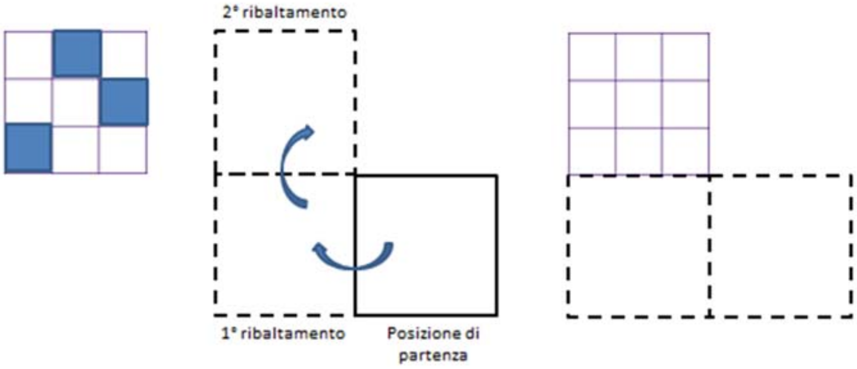

Esercizio 2.

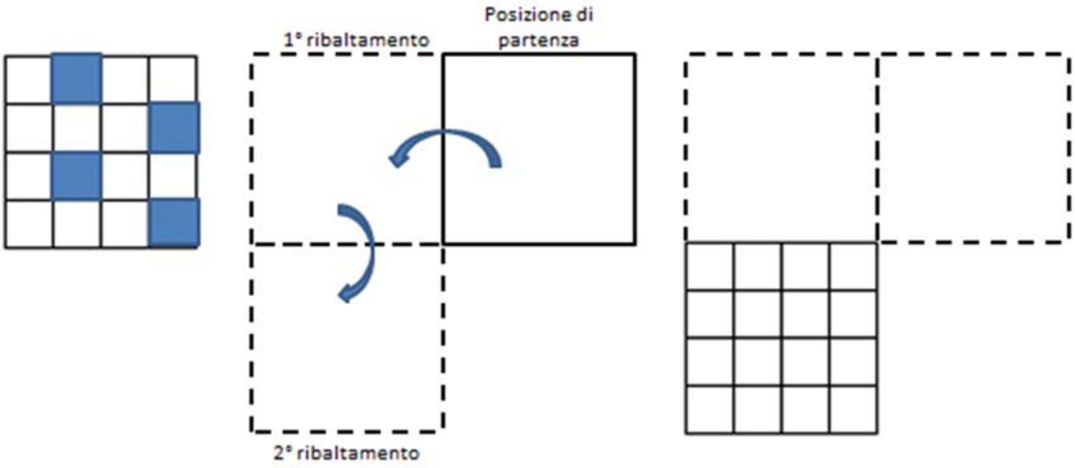

RICOSTRUZIONE DI CUBI (A)

Consegna

Nella parte superiore del foglio è disegnato un cubo tridimensionale. Su ciascuna delle tre facce visibili sono disegnate delle figure (fig. A).
In basso sono proposte delle alternative di sviluppo bidimensionale (fig. B, fig. C) del cubo sopradescritto. Solamente una delle alternative corrisponde al cubo tridimensionale per quanto riguarda il posizionamento delle figure sulle facce.
Selezioni l'opzione che secondo lei mostra il corretto sviluppo bidimensionale del cubo.

fig. A

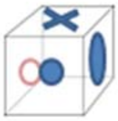

fig. B

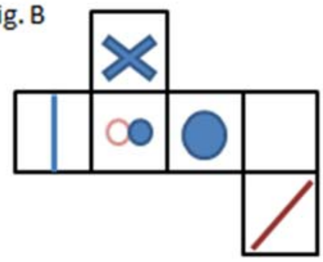

fig. C

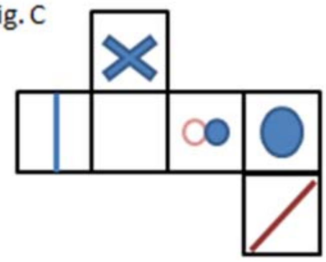

Esercizio 1.

Risposta: _____

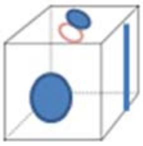

A

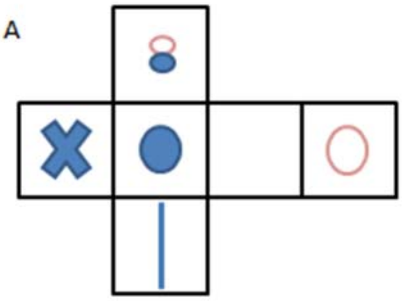

B

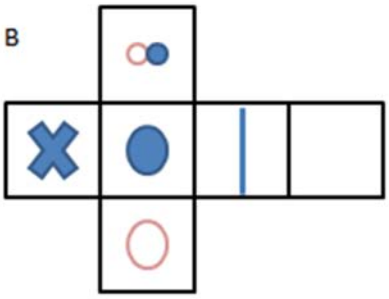

C

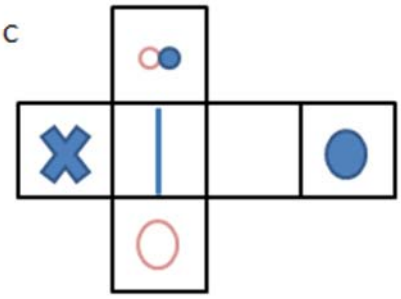

D

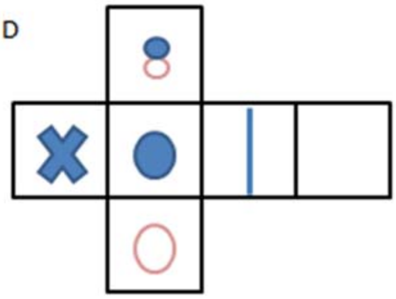

Esercizio 2.

Risposta: _____

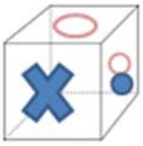

A

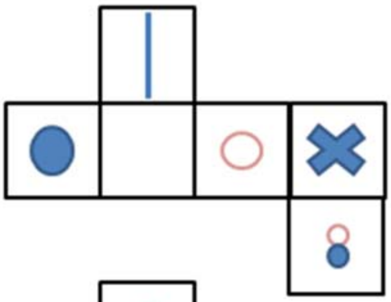

B

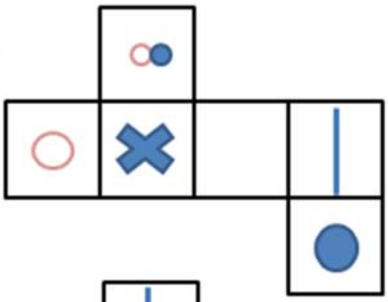

C

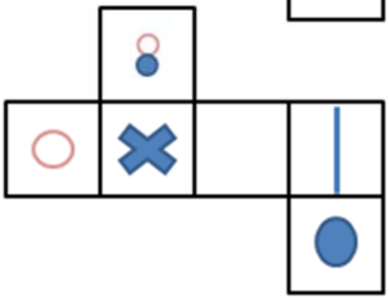

D

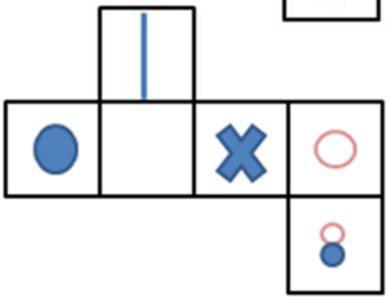

RICOSTRUZIONE DI CUBI (B)

Consegna

Nella parte sinistra del foglio è disegnato lo sviluppo bidimensionale delle sei facce di un cubo (fig. A). Su una faccia è disegnata una freccetta nera e su un'altra faccia una freccetta bianca.
Deve ora cercare di ricostruire mentalmente il cubo nelle tre dimensioni.
Nella parte destra del foglio (fig. B) è riportato un cubo tridimensionale su cui è segnato il punto in cui viene a trovarsi la freccetta nera dopo la ricostruzione del cubo. Indichi su questo cubo il punto in cui, dopo la ricostruzione mentale, si verrà a trovare la freccetta bianca.

fig. A

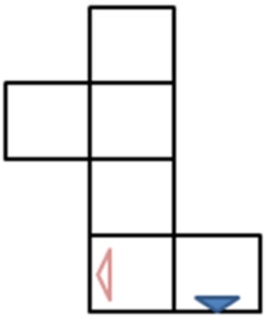

fig. B

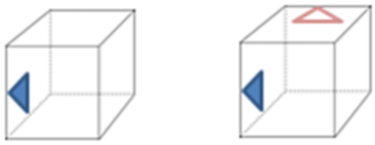

Esercizio 1.

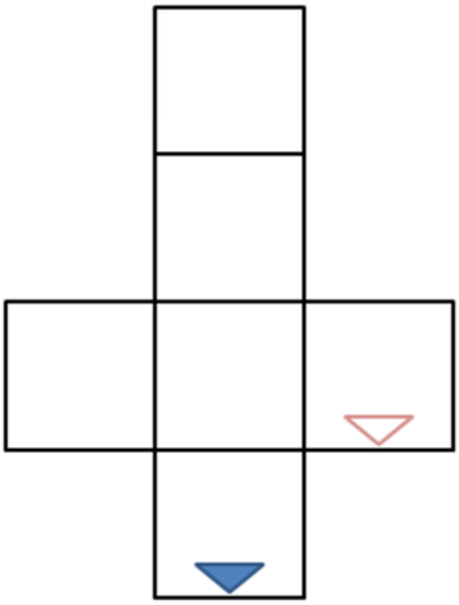

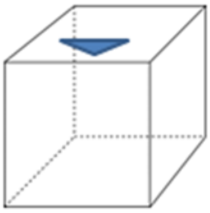

Risposta

Esercizio 2.

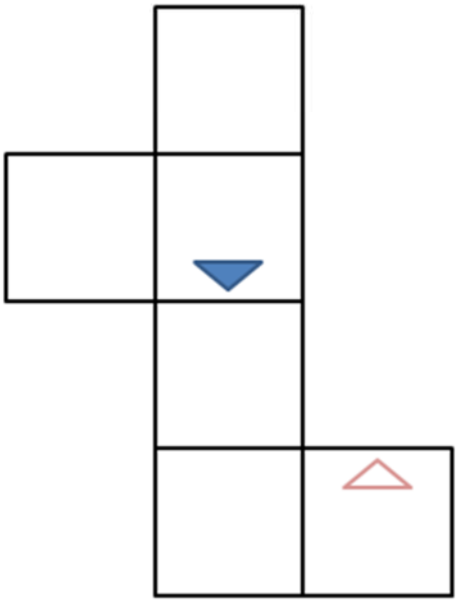

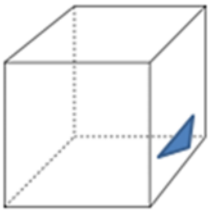

Risposta

Esercizio 3.

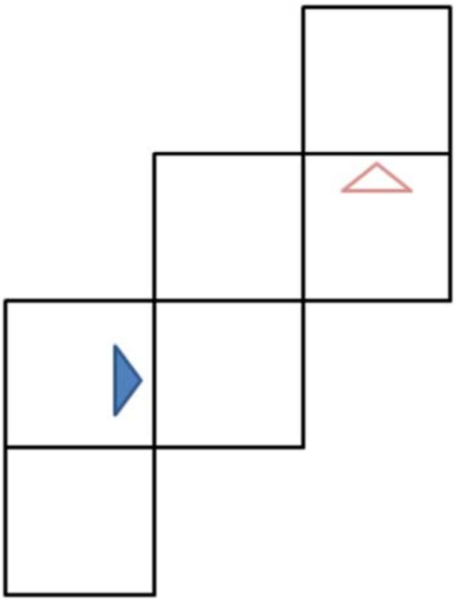

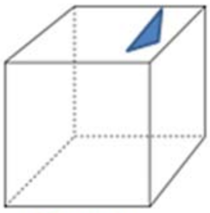

Risposta

CONFRONTO TRA CUBI

Consegna

In alto a sinistra è disegnato un cubo tridimensionale. Sulle tre facce visibili sono disegnate delle figure (fig. A). L'immagine accanto mostra quali sono le altre figure disegnate sul cubo.
A destra si trovano dei cubi tridimensionali. Sulla base del posizionamento delle figure sulle facce, solamente uno di questi corrisponde al cubo sopradescritto ruotato.
Selezioni l'opzione che secondo lei corrisponde al cubo in alto a sinistra ruotato.

Fig. A

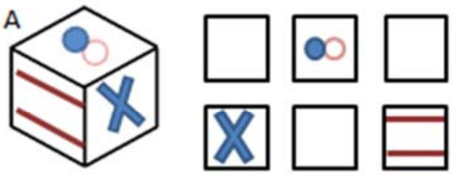

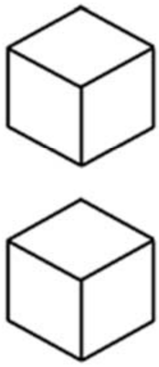

Esercizio 1.

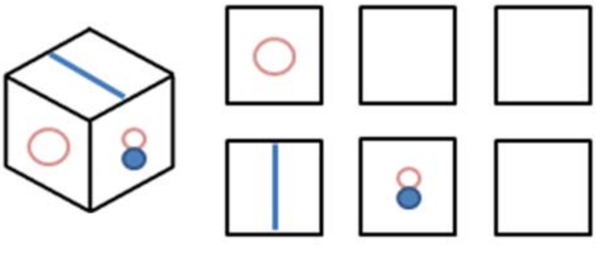

A

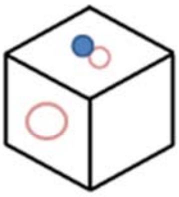

B

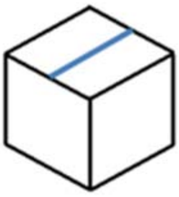

C

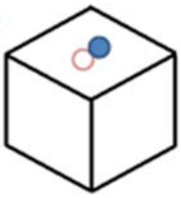

D

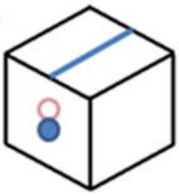

Risposta: _____

Esercizio 2.

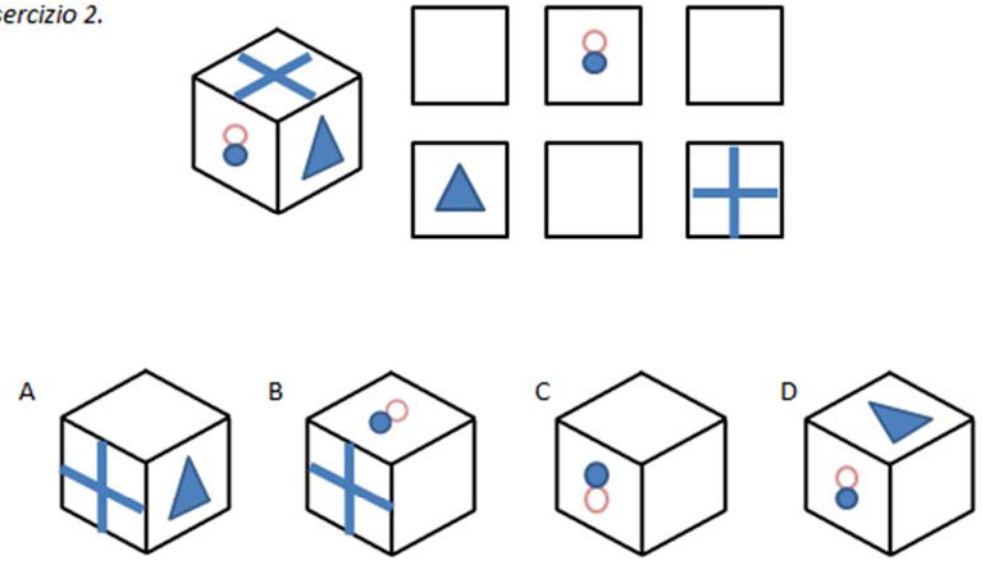

Risposta:_____

Esercizio 3.

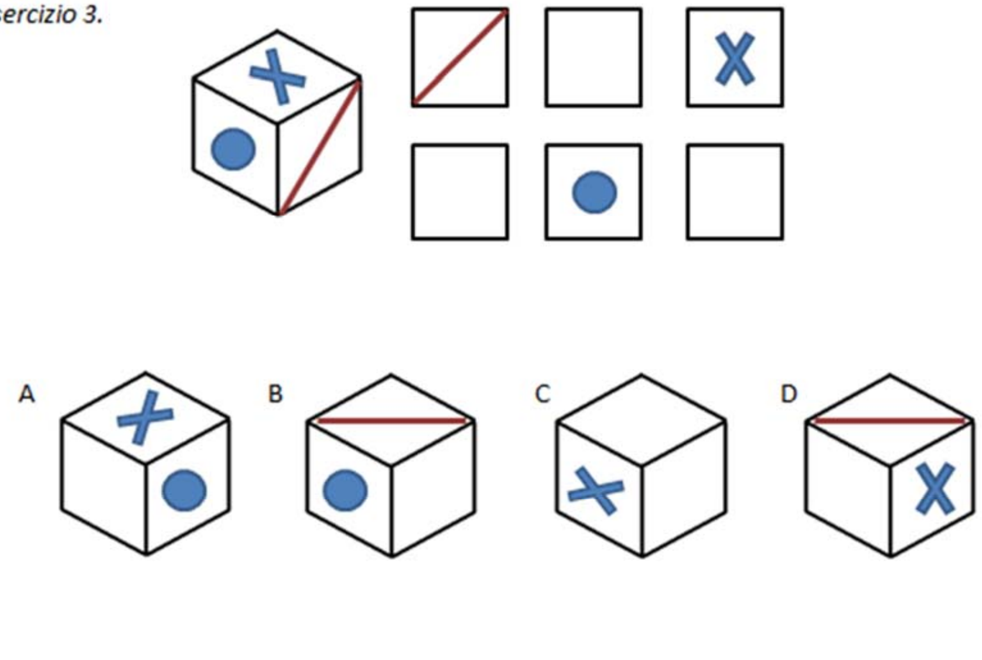

Risposta:_____

ORIENTAMENTO SPAZIALE (A)

Consegna

Le verrà presentata ora un'immagine raffigurante una struttura solida in tre dimensioni, dove il punto X indica la posizione di un osservatore che guarda diritto in fronte a sé (fig. A).
Provi a ruotare l'immagine e ad individuare qual è, tra le alternative proposte (es. fig. B), l'immagine che corrisponde alla prospettiva dell'osservatore.

fig. A

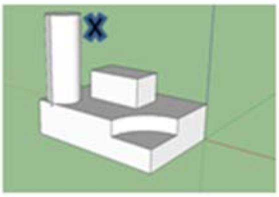

fig. B

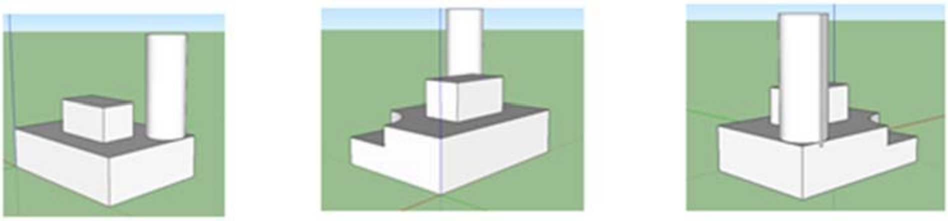

Esercizio 1.

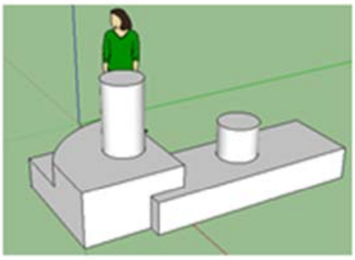

A

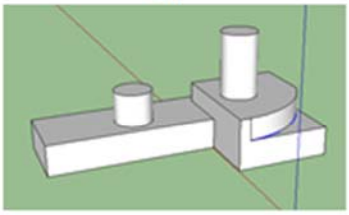

B

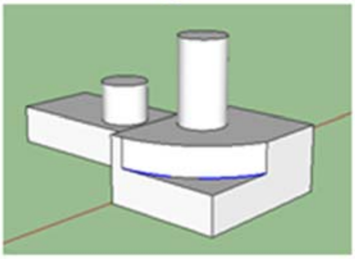

C

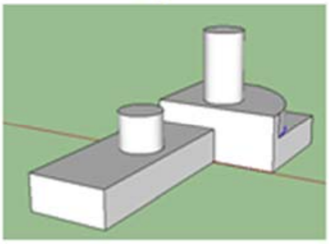

Risposta: _____

Esercizio 2.

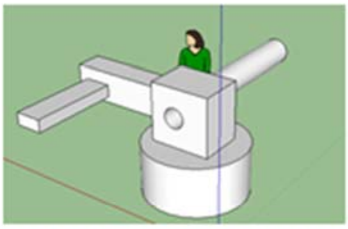

A

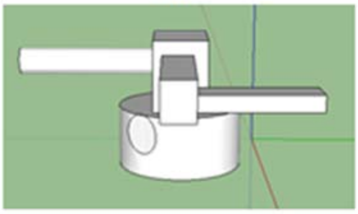

C

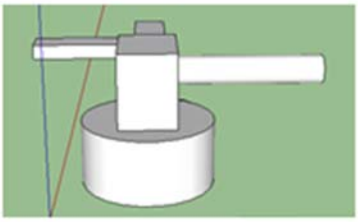

B

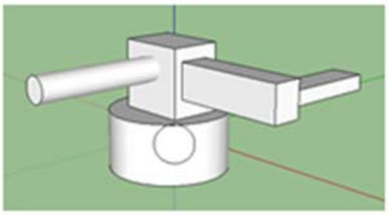

Risposta:_____

Esercizio 3.

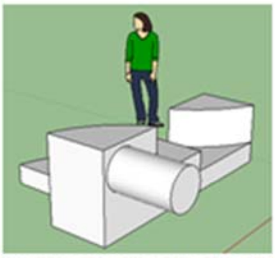

A

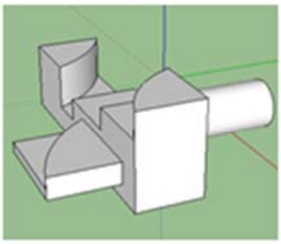

B

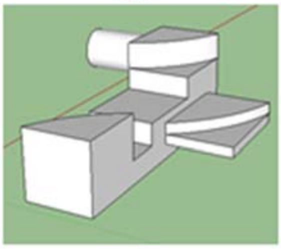

C

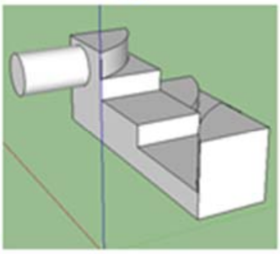

Risposta:_____

ORIENTAMENTO SPAZIALE (B)

Consegna

Le verrà presentata ora un'immagine raffigurante una struttura solida in tre dimensioni, come appare nella prospettiva dell'osservatore che guarda diritto davanti a sé (fig. A).
Osservando il medesimo solido dall'alto (fig. B) segni con una X il corretto posizionamento dell'osservatore nello spazio bianco circostante all'immagine.

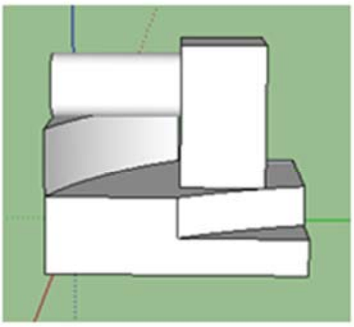

fig. A

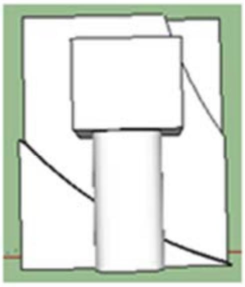

fig. B

Esercizio 1.

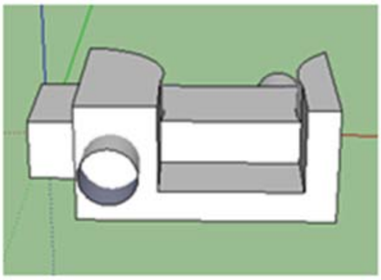

Prospettiva dell'osservatore

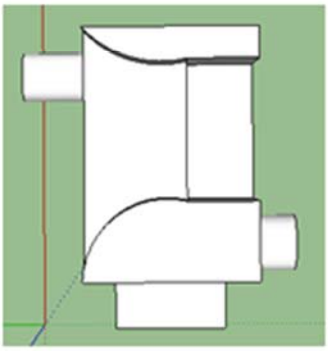

Indichi con una X dove si trova l'osservatore

Esercizio 2.

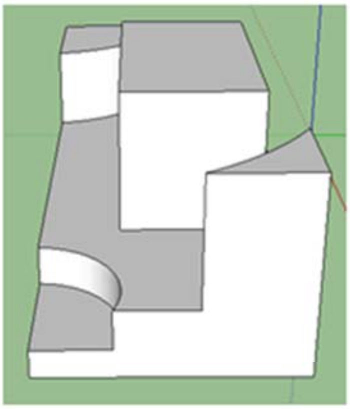

Prospettiva dell'osservatore

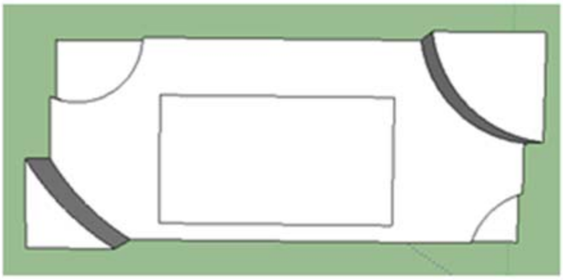

Indichi con una X dove si trova l'osservatore
