# Supplementary material for: The Combined Effect of Acute Interval and Cognitive Training on Visual-Spatial Abilities in Women: Preliminary Insights for Health Promotion
Source: Int J Environ Res Public Health. 2025 Oct 5;22(10):1524. doi: 10.3390/ijerph22101524 (PMC12563624; doi:10.3390/ijerph22101524)
Supplement: Supplementary file 1 [file ijerph-22-01524-s001.zip › S2_Physiological data during trainings.pdf]

S2: Physiological data during trainings

The Table A reports the HR (bpm) during physical and cognitive training. HR in HIIT and LVIT groups was significantly ($p<0.001$) higher during the physical training and during the cognitive training when compared with the COG and CTRL groups.

Table A. HR (bpm) during physical and cognitive training

	**HIIT (N18)**	**LVIT (N18)**	**COG (N18)**	**CTRL (N18)**
Physical training	141.9±17.6[#]	146.4±18.7[#]	73.7±3.1	75.9±1.6
Cognitive training	102.0±4.5[#]	106.1±7.7[#]	71.2±2.8	74.8±2.0

[#] significant difference compared to COG and CTRL ($p < 0.001$). HIIT: High-Intensity Interval Training group; LVIT: Low-Volume Interval Training group; COG: Cognitive Training group; CTRL: Control group.

The Figure A shows the mean HR values of all participants recorded during HIIT, LVIT, COG, and CTRL.

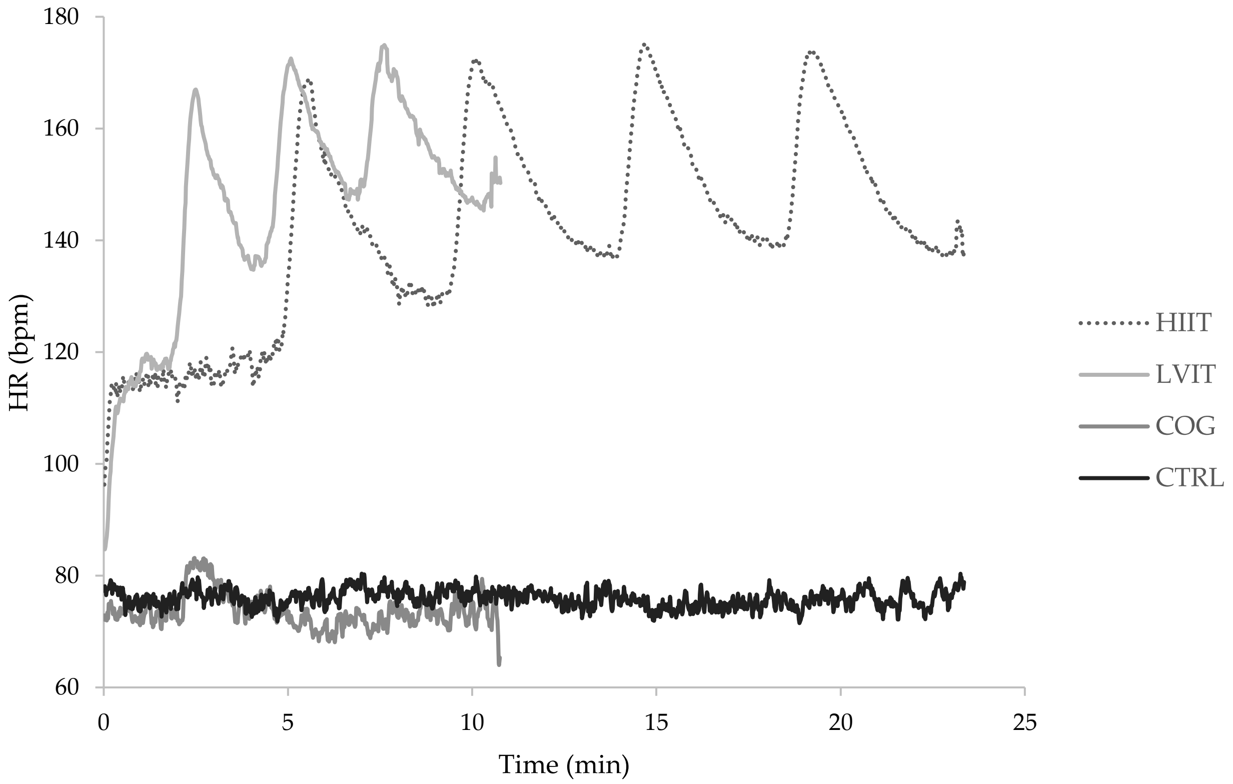

Figure A. HR during physical training

HIIT: High-Intensity Interval Training group; LVIT: Low-Volume Interval Training group; COG: Cognitive Training group; CTRL: Control group

The Figure B shows the mean HR values of all participants recorded during cognitive training.

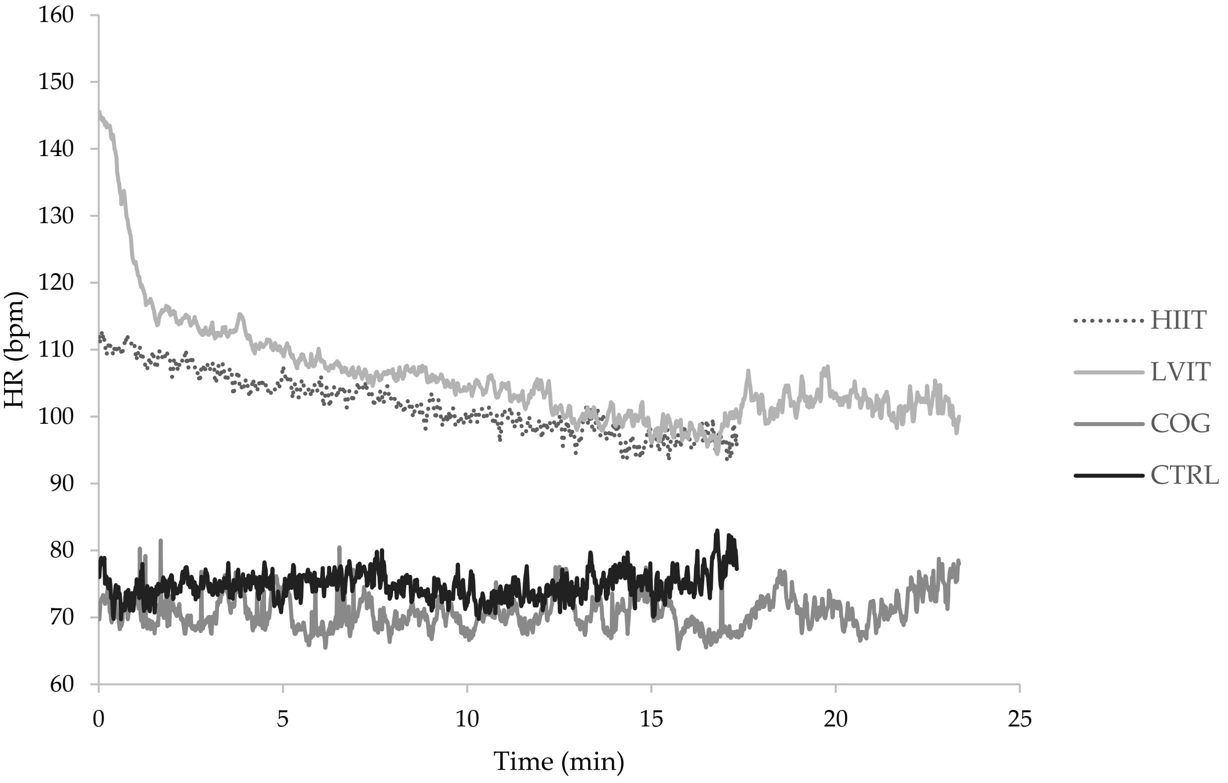

Figure B. HR during cognitive training

HIIT: High-Intensity Interval Training group; LVIT: Low-Volume Interval Training group; COG: Cognitive Training group; CTRL: Control group
